# Reasons for revision are associated with rerevised total knee arthroplasties: an analysis of 8,978 index revisions in the Dutch Arthroplasty Register

**DOI:** 10.1080/17453674.2021.1925036

**Published:** 2021-05-14

**Authors:** Maartje Belt, Gerjon Hannink, José Smolders, Anneke Spekenbrink-Spooren, Berend W Schreurs, Katrijn Smulders

**Affiliations:** a Research Department, Sint Maartenskliniek, Nijmegen; b Interdisciplinary Consortium for Clinical Movement Sciences & Technology (ICMS); c Department of Operating Rooms, Radboud University Medical Center, Radboud Institute for Health Sciences, Nijmegen; d Department of Orthopedics, Sint Maartenskliniek, Nijmegen; e Dutch Arthroplasty Register (Landelijke Registratie Orthopedische Implantaten), ‘s-Hertogenbosch; f Department of Orthopaedics, Radboud University Medical Center, Radboud Institute for Health Sciences, Nijmegen, The Netherlands

## Abstract

Background and purpose — From previous studies, we know that clinical outcomes of revision total knee arthroplasty (rTKA) differ among reasons for revision. Whether the prevalence of repeat rTKAs is different depending on the reason for index rTKA is unclear. Therefore, we (1) compared the repeat revision rates between the different reasons for index rTKA, and (2) evaluated whether the reason for repeat rTKA was the same as the reason for the index revision.

Patients and methods — Patients (n = 8,978) who underwent an index rTKA between 2010 and 2018 as registered in the Dutch Arthroplasty Register were included. Reasons for revision, as reported by the surgeon, were categorized as: infection, loosening, malposition, instability, stiffness, patellar problems, and other. Competing risk analyses were performed to determine the cumulative repeat revision rates after an index rTKA for each reason for revision.

Results — Overall, the cumulative repeat revision rate was 19% within 8 years after index rTKA. Patients revised for infection had the highest cumulative repeat revision rate (28%, 95% CI 25–32) within 8 years after index rTKA. The recurrence of the reason was more common than other reasons after index rTKA for infection (18%), instability (8%), stiffness (7%), and loosening (5%).

Interpretation — Poorest outcomes were found for rTKA for infection: over 1 out of 4 infection rTKAs required another surgical intervention, mostly due to infection. Recurrence of other reasons for revision (instability, stiffness, and loosening) was also considerable. Our findings also emphasize the importance of a clear diagnosis before doing rTKA to avert second revision surgeries.

The number of revision total knee arthroplasties (rTKA) has increased over the past years, and projections predict further increases in the coming decades (Kurtz et al. 2007, Patel et al. 2015, LROI 2019). The outcome of these rTKAs is in general inferior compared with the outcome of the primary total knee arthroplasty (Greidanus et al. 2011, Baker et al. 2012, Nichols and Vose 2016). Evidence suggests that one of the determinants for outcome of rTKA is the indication for the revision. To illustrate, several studies have shown a poor prognosis when the rTKA is performed for infection or stiffness compared with revisions for aseptic loosening (Sheng et al. 2006, Pun and Ries 2008, Baker et al. 2012, Van Kempen et al. 2013, Leta et al. 2015). Poor results were reported in terms of complication rates, patient satisfaction, and survival of the prosthesis. However, the majority of these studies based their findings on small samples, and single-center cohorts.

A repeat revision indicates that either the initial problem was not resolved despite the index revision, or that another problem occurred. Several reasons for a failed index rTKA can be: inaccurate diagnosis, the decision to choose operative versus nonoperative treatment, surgical failure, the occurrence of complications, or insufficient rehabilitation protocols. Insight into whether the reason for index rTKA is related to the same reason for the repeat rTKA might provide a base for improvement of treatment choices in these revision surgeries.

Therefore, we (1) compared the repeat revision rates among the different reasons for index rTKA, and (2) evaluated how often the reason for repeat rTKA was the same as the reason for the index revision.

## Patients and methods

Data was obtained from the Dutch Arthroplasty Register (LROI), which is a nationwide register on all arthroplasties performed in the Netherlands that started in 2007. The data completeness for rTKAs is 97% up to 2018 (LROI 2019). The completeness was first assessed in 2012, yielding 86% coverage. Thus, there is no complete coverage of all rTKAs performed in the Netherlands between 2010 and 2018. All hospitals in the Netherlands report patient characteristics, surgical specifications of each knee arthroplasty procedure, and patient-reported outcomes to the LROI (LROI 2019). To ensure all revision cases were revisions after primary TKA, we retrieved data of all patients who had a primary TKA in the Netherlands between 2007 and 2018. Next, we excluded all cases without rTKA or with an rTKA registration before 2010 due to limited completeness of rTKA before 2010. The first revision after primary TKA was defined as the index rTKA. The second revision after primary TKA was defined as the repeat rTKA. Patients who had received a hinged-type prosthesis as primary implant, or who had a primary TKA performed because of a tumor, were excluded.

Reasons for revision were registered in the LROI as infection, patellar dislocation, patellar pain, wear of the insert, periprosthetic fracture, malalignment, instability, loosening of the femoral component, loosening of the tibial component, loosening of the patellar button, revision after removal of prosthesis, arthrofibrosis, and other reason for revision. Multiple reasons could be reported for one revision procedure by the surgeon. When multiple reasons for revision were registered for one patient, we used a hierarchy tree to define the main reason for the revision. This hierarchy is based on the Australian Orthopaedic Association National Joint Replacement Registry (AOANJRR 2020). The hierarchy was: infection, malposition, loosening (component loosening of femur and/or tibia), patellar problems, instability, stiffness (arthrofibrosis), and other (fracture, wear insert, other non-specified).

An rTKA was defined as a report of any change (insertion, replacement and/or removal) of one or more components of the prosthesis in the register. Time to event was defined as the time between the index revision surgery and repeat rTKA or death. In case of a 2-stage revision (n = 367), we used the re-implantation date as index revision.

The study was conducted and reported according to STROBE guidelines.

### Statistics

The median follow-up time was calculated using reverse Kaplan–Meier. Competing risk analysis was performed to determine the cumulative incidence of repeat revision rates after index rTKA, with death considered as competing event, stratified for the reason of index revision. Log-rank tests were used to test differences in repeat revision rate between the reasons for index revision. To evaluate the probability of having a repeat rTKA for the same reason as the index revision, we conducted a competing risk analysis. In this analysis competing events were a repeat rTKA for any reason other than the reasons for index revision and death. Differences in repeat revision rate were tested with a log-rank test. 95% confidence intervals (CI) were calculated for the cumulative incidences. All analyses were performed using R version 3.6.1 (R Foundation for Statistical Computing, Vienna, Austria) using the packages “rms” and “survival” (Harrell 2020, Therneau 2020).

### Ethics, funding, data sharing, and potential conflict of interest

Ethical approval for the current study was not applicable according to the Dutch Medical Research Involving Human Subjects Act. Data are available from the LROI (Dutch Arthroplasty Register). This study received no funding, and the authors declare that they have no competing interests.

## Results

### Characteristics of index revisions

Between January 2010 and December 2018, a total of 8,868 patients underwent 8,978 index rTKAs as registered in the LROI (110 bilateral rTKA cases). 432 (4%) patients died during the follow-up period. The mean age at the time of the index revision surgery was 67 years (SD 9.6), and 65% were females (Table 1). A patellar problem (n = 2,058, 23%) was the most common reason for index revision; 93% of the index revisions for patellar problems were isolated patellar resurfacings. In 700 rTKAs (8%) the reason for index revision was classified as “other,” and in 354 rTKAs (4%) the reason for revision was not reported.

**Table 1.** Patient characteristics by reason for index rTKA. Values are count (%) unless otherwise specified

Reason for revision **^a^** Patellar								
Factor	Infection (n = 1,538)	Loosening (n = 1,422)	Malposition (n = 1,241)	problems (n = 2,043)	Instability (n = 1,452)	Stiffness (n = 228)	Other (n = 700)	Overall (n = 8,978)
Age, mean (SD)	69 (9.6)	67 (8.9)	66 (9.4)	68 (9.5)	65 (9.5)	64 (9.2)	68 (10.4)	67 (9.6)
Female sex	787 (51)	955 (67)	871 (70)	1,390 (68)	964 (66)	135 (59)	451 (64)	5,787 (65)
Missing	2 (0.1)	1 (0.1)	2 (0.1)	4 (0.2)	3 (0.2)	2 (0.9)	2 (0.3)	17 (0.2)
ASA								
I	157 (10)	158 (11)	176 (14)	224 (11)	204 (14)	38 (17)	95 (14)	1,081 (12)
II	853 (56)	972 (68)	837 (67)	1,439 (70)	989 (68)	154 (68)	445 (64)	5,807 (63)
III–IV	504 (33)	273 (19)	205 (17)	341 (17)	233 (16)	30 (13)	138 (20)	1,780 (20)
Missing	24 (1.6)	19 (1.3)	23 (1.9)	39 (1.9)	26 (1.8)	6 (2.6)	22 (3.1)	310 (3.5)
Diagnosis of primary TKA								
Osteoarthrosis	1,435 (93)	1,344 (95)	1,169 (94)	1,950 (95)	1,350 (93)	212 (93)	655 (94)	8,446 (94)
Osteonecrosis	6 (0.4)	8 (0.6)	2 (0.2)	4 (0.2)	3 (0.2)	0 (0)	2 (0.3)	26 (0.3)
Posttraumatic	37 (2.4)	27 (1.9)	25 (2.0)	32 (1.6)	45 (3.1)	10 (4.4)	11 (1.6)	191 (2.1)
Rheumatoid arthritis	33 (2.1)	19 (1.3)	16 (1.3)	33 (1.6)	27 (1.9)	4 (1.8)	17 (2.4)	151 (1.7)
Inflammatory arthritides	3 (0.2)	0 (0)	0 (0)	0 (0)	2 (0.1)	1 (0.4)	1 (0.1)	7 (0.1)
Other	10 (0.7)	5 (0.4)	8 (0.6)	6 (0.3)	8 (0.6)	1 (0.4)	4 (0.6)	49 (0.5)
Missing	14 (0.9)	19 (1.3)	21 (1.7)	18 (0.9)	17 (1.2)	0 (0)	10 (1.4)	108 (1.2)
Follow-up years, median	3.0	3.2	3.5	3.7	2.9	2.7	3.7	3.4
IQR	1.5–5.2	1.6–5.6	1.8–5.5	1.9–5.8	1.4–4.9	1.5–4.1	1.8–6.3	1.7–5.5

**^a^** Reasons for revision in the table are those from the hierarchy.

### Repeat revision TKA (Table 2)

1,123 repeat rTKAs following the index rTKA were registered. The most common reasons for repeat rTKA were infection (n = 366, 33%), instability (n = 208, 18%), and loosening (n = 195, 17%).

The cumulative repeat revision rate of all index rTKA was 6% (CI 5–6) within 1 year after surgery, and 19% (CI 18–20) within 8 years. A log-rank test showed a statistically significant difference in repeat revision rate between reasons for index revision. The highest cumulative repeat revision rate within 8 years was observed for index revision for infection (28%; CI 25–32) (Figure). Patients revised for instability and stiffness had lower repeat revision rates compared with the infection group. The cumulative repeat revision rate for an index rTKA for instability was 23% (CI 18–28) at 8 years. In rTKAs revised for stiffness the cumulative repeat revision rate was 23% (CI 16–32), the maximum observed follow-up for this group, 6 years after index rTKA. rTKAs revised for loosening, malposition, or patellar problems had the lowest rate of repeat revision surgeries. The cumulative repeat revision rate within 8 years for loosening was 17% (CI 14–20), and for malposition and patellar problems 15% (CI 11–19).

**Figure:**
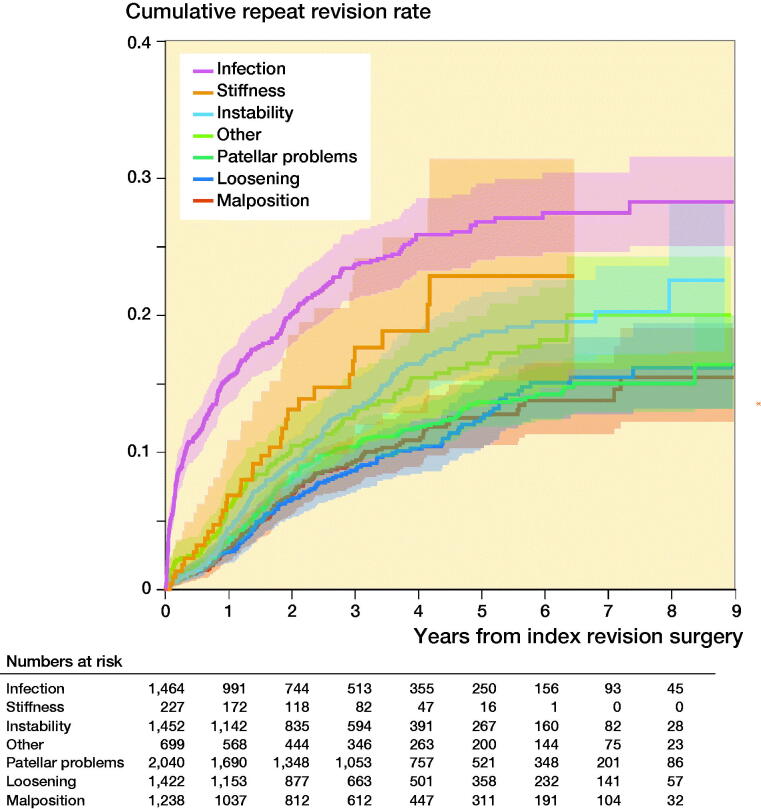
Cumulative repeat revision rate of index rTKA by reason for revision.

### Reason for repeat revision by reason for index revision

In cases index revised for infection who needed repeat rTKA within 8 years, the most common reason for the repeat rTKA was infection (18%; CI 15–21; Table 2). Similar results were observed when an index revision was performed for instability, stiffness, or loosening. The cumulative incidence of a repeat revision for the same reason as the index revision was 8% (CI 6–10) for instability, 7% (CI 3–14) for stiffness, and 5% (CI 4–7) for loosening. See Supplementary data for the cumulative repeat revision rates and specified reason for repeat rTKA.

**Table 2.** Cumulative repeat revision rate after rTKA by reason for revision

	Repeat revision rate (95% CI)		
Factor	at 1 year	at 8 years	at 8 years ^a^
Overall	0.06 (0.05–0.06)	0.19 (0.18–0.20)	–
Infection	0.16 (0.14–0.18)	0.28 (0.25–0.32)	0.18 (0.15–0.21)
Loosening	0.03 (0.02–0.04)	0.16 (0.13–0.19)	0.05 (0.03–0.06)
Malposition	0.03 (0.02–0.04)	0.15 (0.12–0.19)	0.02 (0.01–0.03)
Patellar problems	0.04 (0.03–0.05)	0.15 (0.13–0.17)	0.02 (0.02–0.03)
Instability	0.04 (0.03–0.06)	0.23 (0.17–0.28)	0.07 (0.05–0.09)
Stiffness	0.07 (0.04–0.11)	0.23 (0.15–0.31) ^b^	0.07 (0.03–0.14) ^b^
Other	0.06 (0.04–0.08)	0.20 (0.16–0.24)	0.01 (0.00–0.04)

**^a^** For the same reason as the index revision.

**^b^** At 6-year follow-up.

## Discussion

Poorest outcomes in terms of a repeat rTKA were observed in patients who had had an rTKA for infection. More than 1 in 4 cases revised for infection needed repeat rTKA for any reason; almost 1 in 5 had a repeat rTKA due to a new or recurrent infection, within 8 years after index surgery. The lowest repeat revision rates were observed in index rTKAs for aseptic loosening, malposition, or patellar problems. However, repeat revision rates in these groups were still substantial, with a cumulative repeat revision rate between 15% and 23%. Consistent with infection, in index rTKAs revised for loosening, instability, or stiffness the most prevalent reason for the repeat revision was the same as the index revision.

The most common reason for index rTKA was patellar problems (23%), while in other registries infection and loosening are reported as most common reasons for revision (National Joint Registry 2020). An explanation for this finding may stem from the relatively low percentage of primary TKAs with resurfaced patellae in Dutch clinical practice (18%) compared with most other registries (4–82%) (Fraser and Spangehl 2017). This increases the likelihood that in the case of poor outcomes in non-resurfaced primary TKAs, a first step is to resurface the patella in a reoperation (Teel et al. 2019). Indeed, in our dataset most index rTKAs in patients with patellar problems were isolated resurfacings (> 92%).

A large body of literature has consistently shown that periprosthetic joint infections are difficult to treat (Mortazavi et al. 2011, Kurtz et al. 2018, Leta et al. 2019). Our findings of the repeat revision rate after revision for infection are comparable to the Norwegian Arthroplasty Registry. 5 years after rTKA for infection, 21% of the patients had a repeat rTKA (Leta et al. 2019). The majority of these patients underwent a repeat rTKA due to infection (85 of the 104 repeat revision cases). The large number of infections in index and repeat rTKAs shows that we should keep focusing on the treatment and prevention of joint infections.

It is worth mentioning that more patients revised for infection were classified as ASA class 3 + 4 compared with the other reasons for revision (33% vs. 20% overall). Whether patients with high ASA class are more susceptible to infection, patients with an infection are more likely to receive revision surgery even if they are ASA 3+, or patients with a high ASA class are more likely to need repeat rTKA cannot be concluded from our data.

We observed a higher repeat revision rate after index rTKA for instability and stiffness compared with the NJR (NJR number of subsequent repeat rTKA: 10% after instability, 12% after stiffness) (National Joint Registry 2020). These differences might be explained by the method of reporting the incidence (cumulative incidence versus percentage by the NJR), due to different definitions of the indications, or due to the willingness to reoperate. Nonetheless, the NJR reported that instability, infection, and stiffness are more common indications for repeat rTKA than for index rTKA, which corresponds to the results of our study. The NJR hypothesizes that repeat rTKA for instability, infection, and stiffness reflects the complexity and soft tissue element that contribute to the outcome of rTKA (National Joint Registry 2020). The latter is consistent with the generally poor results that are reported after rTKA for stiffness and instability (Kim et al. 2010, Malviya et al. 2012, Luttjeboer et al. 2016).

Lowest repeat revision rates were found in patients revised for loosening, malposition, and patellar problems. This is in line with multiple previous studies (Sheng et al. 2006, Baker et al. 2012, Leta et al. 2015). However, the majority of the index revisions for patellar problems were isolated patellar resurfacings (93%). In 10% of the cases this isolated patellar resurfacing was followed by a subsequent repeat revision for amongst other causes infection, malposition, and instability. This suggests that the initial patellar resurfacing did not address the original failure diagnosis or induced a new one.

Our findings should be regarded in the context of a number of strengths and limitations. The use of nationwide registry data has benefits, including the large sample size and high generalizability. Another strength is we accounted for death as competing event in the survival analysis of revision TKA, which potentially provides a more accurate estimate of the repeat revision rate than Kaplan–Meyer analysis. Also, we did not limit the inclusion of rTKAs to patients who had a primary TKA for osteoarthritis (OA), to make the results generalizable to all revision TKA patients. We performed an additional analysis where we included only patients with OA. This additional analysis showed cumulative repeat revision rates similar to those reported in the current manuscript.

A limitation of our analysis method is that a subject can only have 1 reason for revision in the analysis, while multiple reasons were reported in some cases. Therefore, we used a hierarchy in the reasons for revision to rank cases with more than 1 reason for revision. A sensitivity analysis showed this resulted in slightly different cumulative repeat revision rate estimates (see Supplementary data). Second, to ensure that all cases in our study were the first revision after primary TKA, we included only cases with the primary TKA registered. As a consequence, the follow-up time of the patients was limited. Complications that often present shortly after surgery, such as infection, are therefore better represented in the data compared with long-term complications such as loosening, resulting in higher repeat rTKA estimates for the short-term reasons for revision compared with the reasons that present long term. Thus, the repeat revision surgeries were mostly due to short- to mid-term complications. Third, the reason for revision was registered by orthopedic surgeons who may use different interpretations of the definitions for the reasons. Another limitation related to the registry data is that the registry forms are filled in once, directly following the surgery. A (suspected) infection might not be proven at that point; thus cases of infection might still be underreported despite the already high proportion of revision due to infection (Gundtoft et al. 2016, Afzal et al. 2019). Also, the registry does not have complete coverage of all primary and rTKA procedures performed in the Netherlands between 2007 and 2018. Fourth, we did not correct for correlated bilateral cases in the analysis, while the methods of our statistical analysis do assume independent observations, although previous studies have shown bilateral surgeries do not introduce significant dependency problems in register studies (Robertsson and Ranstam 2003, Park et al. 2010). Finally, we acknowledge the ongoing discussion of survival analysis in arthroplasty registers considering ease of interpretation versus accuracy of survival. Kaplan–Meier and competing risk analysis each have their advantages and disadvantages. However, we decided to report cumulative incidences of repeat rTKA.

In conclusion, the reason for index revision seems to be associated with the incidence of repeat rTKA at 8 years’ follow-up. Poorest outcomes were found for rTKA for infection: more than 1 in 4 infection rTKAs required another surgical intervention, often due to a new or persistent infection. Recurrence of other reasons for revision (instability, stiffness, and loosening) was also considerable. This study confirms the complex treatment to manage periprosthetic infections. Our findings also emphasize the importance of a clear diagnosis before doing rTKA to avert second revision surgeries.

## Note

Please note that there is a relatively large difference in numbers of index rTKAs included in this study and reported in the annual report of the LROI. This difference is due to a difference in selection of patients. In the annual report of the LROI, 2-stage revisions and isolated patellar resurfacing revisions were not included. These cases are, however, included in this study. Also, in the present study the selection period was limited to 2010–2018.

## Supplementary Material

Supplemental Material

## Supplementary data

The cumulative repeat revision rates per reason for repeat rTKA and the sensitivity analysis are available as supplementary data in the online version of this article, http://dx.doi.org/10.1080/17453674.2021.1925036
