# Supplementary material for: Reasons for revision are associated with rerevised total knee arthroplasties: an analysis of 8,978 index revisions in the Dutch Arthroplasty Register
Source: Acta Orthop. 2021 May 14;92(5):597–601. doi: 10.1080/17453674.2021.1925036 (PMC8519540; doi:10.1080/17453674.2021.1925036)
Supplement: Supplemental Material [file IORT_A_1925036_SM2318.pdf]

Supplementary data

A. Cumulative repeat revision rate after index rTKA by reason for revision, specified by reason for repeat revision

Reason for index rTKA	Infection	Loosening	Malposition	Patellar problems	Instability	Stiffness	Other
	Reason for repeat revision (95% CI) rate at 8 years						
Infection	0.18 (0.15–0.21)	0.03 (0.02–0.05)	0.02 (0.01–0.03)	0.02 (0.01–0.03)	0.02 (0.01–0.04)	0.00 (0.00–0.01)	0.01 (0.00–0.01)
Loosening	0.03 (0.02–0.05)	0.05 (0.03–0.06)	0.02 (0.01–0.04)	0.02 (0.01–0.03)	0.02 (0.01–0.04)	0.01 (0.00–0.01)	0.01 (0.00–0.04)
Malposition	0.02 (0.01–0.04)	0.03 (0.02–0.04)	0.02 (0.01–0.03)	0.03 (0.02–0.06)	0.03 (0.02–0.06)	0.01 (0.00–0.02)	0.01 (0.01–0.03)
Patellar problems	0.02 (0.01–0.03)	0.03 (0.02–0.04)	0.03 (0.02–0.05)	0.02 (0.02–0.03)	0.03 (0.02–0.05)	0.01 (0.00–0.01)	0.01 (0.00–0.01)
Instability	0.05 (0.02–0.12)	0.03 (0.02–0.05)	0.02 (0.01–0.04)	0.04 (0.03–0.06)	0.07 (0.05–0.09)	0.00 (0.00–0.01)	0.01 (0.00–0.02)
Stiffness [a]	0.04 (0.02–0.07)	0.02 (0.01–0.08)	0.05 (0.02–0.10)	0.03 (0.01–0.13)	0.01 (0.00–0.05)	0.07 (0.03–0.14)	0.01 (0.00–0.05)
Other	0.04 (0.03–0.06)	0.03 (0.02–0.06)	0.03 (0.02–0.05)	0.03 (0.01–0.05)	0.04 (0.02–0.06)	0.01 (0.00–0.04)	0.01 (0.00–0.04)

[a] At 6-year follow-up.

B. Sensitivity analysis hierarchy — a limitation of our main analysis method is that a subject can have only one reason for revision in the analysis, while multiple reasons were reported in some cases. Therefore, we used a hierarchy in the reasons for revision to rank cases with more than one reason for revision. To test the effect of that hierarchy, we performed a sensitivity analysis. The first column is the cumulative repeat revision rate by reason for index rTKA, as reported in the manuscript. In the second column, we did not use a hierarchy in the reasons for revision, but instead conducted a separate competing risk analysis for each of the different reasons for index rTKA. As a result, patients with multiple reported reasons for revision are represented more than once.

Factor	Repeat revision rate at 8 years (95% CI) with hierarchy	without hierarchy
Overall	0.19 (0.18–0.20)	–
Infection	0.28 (0.25–0.32)	0.28 (0.25–0.32)
Malposition	0.15 (0.12–0.19)	0.16 (0.12–0.19)
Loosening	0.16 (0.13–0.19)	0.19 (0.16–0.21)
Patellar problems	0.15 (0.13–0.17)	0.16 (0.14–0.18)
Instability	0.23 (0.17–0.28)	0.22 (0.18–0.26)
Stiffness [a]	0.23 (0.15–0.31)	0.22 (0.16–0.29)
Other	0.20 (0.16–0.24)	0.20 (0.17–0.23)

[a] At 6-year follow-up
